# Decreased Functional Brain Connectivity in Adolescents with Internet Addiction

**DOI:** 10.1371/journal.pone.0057831

**Published:** 2013-02-25

**Authors:** Soon-Beom Hong, Andrew Zalesky, Luca Cocchi, Alex Fornito, Eun-Jung Choi, Ho-Hyun Kim, Jeong-Eun Suh, Chang-Dai Kim, Jae-Won Kim, Soon-Hyung Yi

**Affiliations:** 1 Melbourne Neuropsychiatry Center, Department of Psychiatry, University of Melbourne and Melbourne Health, Parkville, Victoria, Australia; 2 Florey Institute of Neuroscience and Mental Health, Parkville, Victoria, Australia; 3 Division of Child and Adolescent Psychiatry, Department of Psychiatry, College of Medicine, Seoul National University, Seoul, Republic of Korea; 4 Queensland Brain Institute, University of Queensland, Brisbane, Australia; 5 Centre for Neural Engineering, University of Melbourne, Parkville, Victoria, Australia; 6 NICTA Victorian Research Laboratory, University of Melbourne, Parkville, Victoria, Australia; 7 Department of Child Development and Family Studies, College of Human Ecology, Seoul National University, Seoul, Republic of Korea; 8 Interdisciplinary Program (Early Childhood Education Major), College of Education, Seoul National University, Seoul, Republic of Korea; 9 Center for Campus Life & Culture, Seoul National University, Seoul, Republic of Korea; 10 Department of Education (Educational Counseling Major), College of Education, Seoul National University, Seoul, Republic of Korea; Institute of Psychology, Chinese Academy of Sciences, China

## Abstract

**Background:**

Internet addiction has become increasingly recognized as a mental disorder, though its neurobiological basis is unknown. This study used functional neuroimaging to investigate whole-brain functional connectivity in adolescents diagnosed with internet addiction. Based on neurobiological changes seen in other addiction related disorders, it was predicted that connectivity disruptions in adolescents with internet addiction would be most prominent in cortico-striatal circuitry.

**Methods:**

Participants were 12 adolescents diagnosed with internet addiction and 11 healthy comparison subjects. Resting-state functional magnetic resonance images were acquired, and group differences in brain functional connectivity were analyzed using the network-based statistic. We also analyzed network topology, testing for between-group differences in key graph-based network measures.

**Results:**

Adolescents with internet addiction showed reduced functional connectivity spanning a distributed network. The majority of impaired connections involved cortico-subcortical circuits (∼24% with prefrontal and ∼27% with parietal cortex). Bilateral putamen was the most extensively involved subcortical brain region. No between-group difference was observed in network topological measures, including the clustering coefficient, characteristic path length, or the small-worldness ratio.

**Conclusions:**

Internet addiction is associated with a widespread and significant decrease of functional connectivity in cortico-striatal circuits, in the absence of global changes in brain functional network topology.

## Introduction

Internet addiction has been increasingly recognized both in public and the scientific community worldwide [1], though it is a relatively new condition and its psychopathological characteristics and neurobiological mechanisms remain poorly understood. Recent neuroimaging studies have reported significant changes in brain function and structure associated with internet addiction. The majority of these studies utilized functional magnetic resonance imaging (fMRI) performed during tasks, and have implicated frontal and striatal regions and, to a lesser extent, the parietal and insula cortex [2]–[5]. Additionally, using positron emission tomography (PET), Kim et al. (2011) found decreased dopamine D2 receptor availability in bilateral caudate and right putamen [6], and Hou et al. (2012) using single photon emission computed tomography (SPECT) found decreased striatal dopamine transporter expression level in adults with internet addiction [7]. These findings accord with current theoretical models of addiction disorders, comprising not only substance addiction but also behavioral addiction (e.g., pathological gambling), which emphasize pathology of fronto-striatal circuitry [8], as well as the insula [9], [10]. Structural MRI studies by Zhou et al. (2011) and Yuan et al. (2011) have together suggested gray matter abnormalities in brain regions including the prefrontal cortex [11], [12], and a diffusion-tensor imaging study by Lin et al. (2012) reported widespread white matter abnormalities in adolescents with internet addiction [13]. Lastly, Liu et al. (2010) found altered regional homogeneity in internet addiction [14], which is to our knowledge the only resting-state fMRI finding in the literature regarding this disorder [15]. The authors investigated the temporal homogeneity in the Blood-Oxygen-Level-Dependent (BOLD) signal of each voxel with those of its nearest 26 neighbor voxels in a voxel-wise way.

Resting-state fMRI is a relatively new imaging technique for investigating inter-regional correlations of spontaneous brain activity, recorded as someone lies quietly in the scanner without being engaged in a specific task [16]. The approach provides a robust method for mapping well-defined functional systems [17], [18]. Resting-state measures are reliable [19], [20], under genetic control [21]–[23], and thought to index an intrinsic property of brain functional organization [24], subject to certain caveats [25]. In combination with graph theoretic techniques, resting-state fMRI offers a powerful means for investigating the large-scale organization of brain functional dynamics and its disruption in psychopathological conditions [26].

In this study, we used resting-state fMRI data to map differences in functional connectivity between a comprehensive set of 90 distinct cortical and subcortical brain regions in healthy individuals and adolescents with internet addiction, focusing on individuals engaging in excessive online gaming among the proposed subtypes of this disorder [27]. We also conducted an analysis of network topological disturbances [28] to assess whether any between-group differences in connectivity strength were further associated with a global reconfiguration of functional interactions [26], as has been reported in many other psychiatric disorders [29], [30].

Based on previous structural and functional neuroimaging findings in internet addiction [3], [4], [6], [7], [15], along with the established theoretical models of substance addiction disorders [8], [9], we hypothesized that adolescents with internet addiction would show altered inter-regional connectivity between frontal and striatal regions, with a possible further involvement of parietal cortex and insula.

## Materials and Methods

### Ethics Statement

This study was approved by the institutional review board for human subjects at the Seoul National University. All the adolescents and their parents provided written informed consent prior to study entry. The study was conducted in accordance with the Declaration of Helsinki.

### Participants

Twelve right-handed male adolescents with internet addiction and 11 right-handed and gender-matched [31] healthy controls participated in this study. The diagnosis of internet addiction was established using the Young Internet Addiction Scale (YIAS), which consists of 20 items, each based on a 5-point Likert scale evaluating the degree of problems caused by internet use [32], and the Kiddie-Schedule for Affective Disorders and Schizophrenia-Present and Lifetime Version (K-SADS-PL), a semi-structured diagnostic interview tool with established validity and reliability, which enabled us to exclude other psychiatric disorders [33], [34]. Participants with internet addiction were confined to those reporting to have experienced typical components of addiction (i.e., tolerance, withdrawal, preoccupation with playing online games, repeated unsuccessful attempts to reduce or stop online gaming, negatively influenced mood when attempting to reduce online gaming, and neglecting important relationships or activities because of online games) [35], [36]. All participants with internet addiction reported excessive online gaming among the proposed subtypes of this disorder. The same instruments were applied when recruiting healthy adolescents. Demographic information and intelligence quotient (IQ) of all the participants were also assessed (see Table 1).

**Table 1:** Demographic and clinical characteristics of the participants.

	Internet	Control	P-value
Age (years)	13.41±2.31	14.81±0.87	0.071
Gender (male)	12 (100%)	11 (100%)	N/A
IQ	102.83±16.41	109.63±9.87	0.247
YIAS	57.00±17.39	38.36±7.31	0.004

IQ, intelligence quotient; YIAS, Young Internet Addiction Scale

### Data Acquisition and Image Processing

Resting-state fMRI images were acquired on a 3T Siemens scanner (Siemens Magnetom Trio Tim Syngo MR B17, Germany) with the following parameters: repetition time (TR) 2700 ms; echo time (TE) 30 ms; acquisition matrix 64×64; field of view (FOV) 192×192 mm^2^; flip angle 90°; voxel size 3.0 mm×3.0 mm×3.0 mm; slices 40. The total time of the acquisition was 6 min 45 sec. A head coil was used and head motion was minimized by filling the empty space around the head with sponge material and fixing the lower jaw with a tape.

The preprocessing of fMRI images was conducted using the Data Processing Assistant for Resting-State fMRI (DPARSF) [37], which is based on Statistical Parametric Mapping (SPM8) and Resting-State fMRI Data Analysis Toolkit (REST). The first 5 images in each subject were discarded, and the remaining 145 images were corrected for slice timing and realigned to the first volume in order to correct for motion artifacts. All participants showed less than 0.5 mm of displacement and 0.5° of rotation in their 6 head motion parameters. In addition, the two groups were not significantly different (*p*<0.05) in the four head motion parameters recently suggested by Van Dijk et al. [38]: i.e., mean head displacement (internet addiction: 0.04±0.01 mm, control: 0.04±0.01 mm), maximum head displacement (internet addiction: 0.18±0.14 mm, control: 0.17±0.07 mm), number of micro (>0.1 mm) movements (no more than 2 for all participants, except for two individuals in internet addiction group having 5 and 6 micro movements), and head rotation (internet addiction: 0.04±0.01°, control: 0.04±0.00°). Before spatial normalization, an age- and gender-matched brain template was created based on data from the NIH MRI Study of Normal Brain Development, using Template-O-Matic [39]. Our fMRI images were normalized using this customized template and smoothed with a full-width half-maximum Gaussian kernel of 6 mm. The data were then detrended and low frequency fluctuations (0.01–0.08 Hz) were filtered in order to detect signals from gray matter and reduce the effect of noise. Six head motion parameters, white matter signals, and cerebrospinal fluid signals were regressed from the filtered BOLD signal. Finally, the residuals of this regression were extracted from 90 brain regions (nodes) based on the Automated Anatomical Labeling (AAL) atlas [40], and pair-wise associations were calculated resulting in a 90 by 90 connectivity matrix per each subject. Pearson's correlation coefficient (zero lag) was used to quantify each pair-wise association. Note that the global signal was not included as a nuisance covariate, ensuring the proportion of negative correlations was minimal.

### Data Analysis

The network-based statistic (NBS) [41], [42] was used to identify regional brain networks showing a significant between-group difference in inter-regional functional connectivity. Specifically, a t-test was performed to test for a between-group difference in the correlation coefficient at each of the 90×(90-1)/2 = 4005 unique regional pairings. Interconnected networks, formally known as *graph components*, were then identified among the connections with a t-statistic exceeding a threshold of t = 3.0. A family-wise error (FWE)-corrected p-value was calculated for the size of each resulting component using permutation testing (20000 permutations). Each permutation involved randomly shuffling the group labels and identifying the size of the *largest* interconnected network, thereby yielding an empirical null distribution of maximal component sizes [43]. A FWE-corrected p-value was estimated for each interconnected network as the proportion of permutations that yielded a larger interconnected network, or one of equal size. The two alternative hypotheses (addiction > controls and addiction < controls) were evaluated independently. All these steps were performed using the NBS software package, which is freely distributed as part of the Brain Connectivity Toolbox (http://www.brain-connectivity-toolbox.net/) or NITRC (http://www.nitrc.org/projects/nbs/). To assess the reproducibility of any significant findings to alternative atlases [44], the above analysis was repeated separately with the AAL atlas substituted with two alternative atlases for parcellating the cortex into non-overlapping regions; namely, the Montreal Neurological Institute (MNI) structural atlas (http://www.fmrib.ox.ac.uk/fsl/data/atlas-descriptions.html) and a random parcellation comprising 120 regions [45]. The MNI atlas is a coarse parcellation representing eight anatomical regions per cerebral hemisphere, thereby facilitating characterization of inter-lobar connectivity.

Next, we tested for between-group differences in key graph-based network measures [28]; namely, the average clustering coefficient, characteristic path length and small-worldness ratio. Interpretation of these measures in terms of brain complexity and organization can be found in much recent literature [26], [30], [46]–[49]. The connectivity matrices were first binarized with respect to a set of fixed connection densities, ranging from 10% to 30% [28]. Network measures were calculated at each density using the appropriate function provided in the Brain Connectivity Toolbox. The clustering coefficient and characteristic path length were normalized with respect to an ensemble of 20 random networks generated using the Maslov-Sneppen rewiring algorithm [50]. Between-group differences were then assessed at each density using a two-sided t-test.

Finally, the clustering coefficient and path length were calculated locally for each of the 90 regions. A two-sided t-test was also used to test for between-group differences in these region-specific measures. The false discovery rate (FDR) [51] was used to correct for multiple comparisons across the family of network densities and regions.

## Results

### Participant Characteristics

All participants were right-handed males. No significant difference was found in age and IQ between the two groups, and YIAS score was significantly higher in internet addiction group (Table 1).

### Group Differences in Functional Connectivity

**Figure 1:**
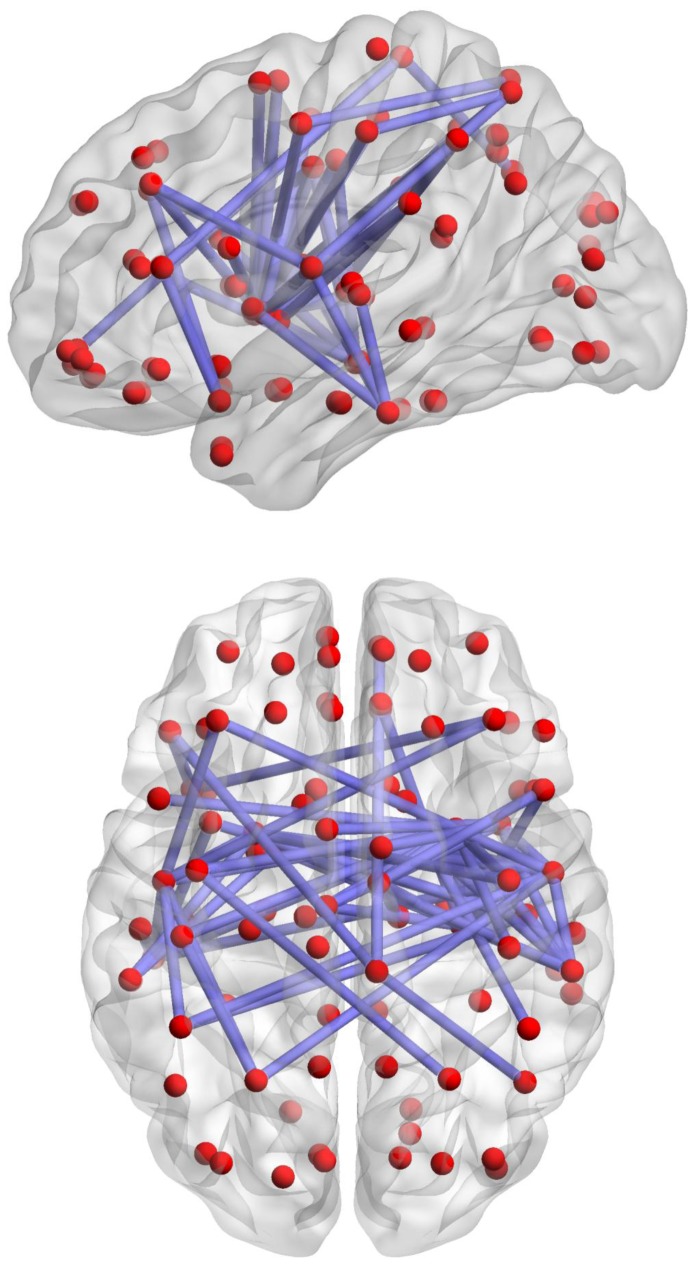
Network of decreased brain functional connectivity in adolescents with internet addiction. Red dots represent stereotactic centroids of brain regions (nodes) defined by Automated Anatomical Labeling (AAL) atlas, and blue lines represent suprathreshold links (t = 3.0) comprising the affected network identified with the network-based statistic (NBS) (*p*<0.05, component-wise corrected).The axial view illustrates the involvement of interhemispheric connections (i.e., connections crossing between the right and left hemisphere). The sagittal view illustrates the involvement of frontal, temporal, and parietal lobes in the affected network.

The NBS identified a single network showing significantly (*p*<0.05, FWE-corrected) decreased connectivity in adolescents with internet addiction compared to controls. This affected network comprised 59 links, involving 38 different brain regions (Figure 1). The network was broadly replicated when the AAL atlas was substituted with two alternative atlases for parcellating the cortex into non-overlapping regions (see Figure S1). Despite considerable variation in the total number of regions comprising these atlases (AAL: 90, MNI: 16, random: 120), remarkable consistency was evident in the overall network structure. Network size expectedly increased with atlas resolution (i.e., total number of regions), giving rise to a more complex configuration. However, the cortical and sub-cortical regions (and corresponding lobes) implicated were broadly replicated across the three atlases. Figures were visualized with the BrainNet Viewer (http://www.nitrc.org/projects/bnv/).

**Figure 2:**
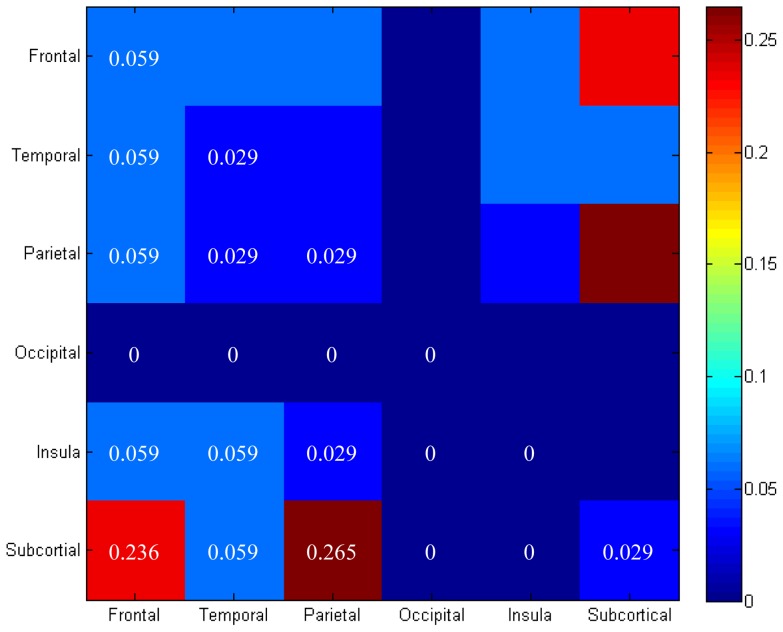
Proportion of connections affected in internet addiction linking distinct pairs of broad cerebral divisions. The number of links involving each pair of divisions is normalized by total number of pair-wise links.Note that the hippocampus, globus pallidus, and putamen were assigned to the subcortical category, and the anterior cingulate gyrus was assigned to the frontal category. The amygdala and caudate nucleus were not included in the disrupted network and thus there was no need to assign these regions to a lobe. The putamen, bilaterally, was the most extensively involved subcortical region, showing decreased connections with all three major cerebral lobes involved.

Following Fornito et al. [52], AAL regions were categorized into corresponding major lobes (e.g., frontal, temporal, parietal) and the proportion of connections linking these large-scale divisions was quantified for each pair of lobes. Fronto-temporo-parietal connections were found to be affected, but the occipital lobe was not included in the affected network. The majority of connections that were reduced in the internet addiction group involved links between subcortical regions and frontal (∼24%) and parietal (∼27%) cortices (Figure 2). To better understand which subcortical regions may be contributing to this finding, we examined the connectivity between each cortical lobe and each subcortical region separately in the NBS network (Figure S2). This analysis revealed that the subcortical regions included hippocampus, globus pallidus, and putamen. The amygdala and caudate nucleus were not included in the affected network. Bilateral putamen was the most extensively involved subcortical region, showing decreased connections with all three major cerebral lobes involved. This pattern was replicated using the MNI atlas, from which the resulting network only included putamen and insula, in addition to frontal, parietal, and temporal lobes; caudate nucleus and occipital lobe were not included in the affected network.

We did not identify any network with increased connectivity in the internet addiction group. No significant correlation was found between functional connectivity in the identified network and YIAS score, either in the internet addiction group or in controls.

### Group Differences in Network Topology

**Figure 3:**
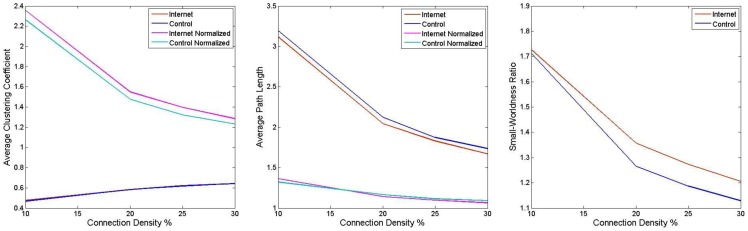
Small-world parameters of brain functional connectivity in adolescents with internet addiction.

No between-group difference was noted in the average clustering coefficient, the characteristic path length or the small-worldness ratio at any of the network densities investigated (*p*<0.05, FDR-corrected) (Figure 3). Additionally, no between-group difference in the corresponding local (region-specific) measures survived FDR correction for multiple comparisons. Applying a less stringent false positive correction *p*<(1/90) = 0.011 [53] in order to explore trend level effects yielded between-group differences in the local clustering coefficient and the local path length appearing predominantly in the occipital lobes (Tables S1 and S2).

## Discussion

Evidence of decreased brain functional connectivity was found in adolescents with internet addiction. Consistent with current models emphasizing the role of cortico-subcortical pathology in addiction [54], 24% of the connections in the altered network differentiating addicted individuals and healthy controls involved links between frontal and subcortical regions. An additional 27% linked subcortical and parietal areas, with more limited evidence for involvement of the insula, again consistent with recent evidence of an involvement of these regions in addiction. Note that our analysis provides a stringent test of cortico-subcortical models of addiction, as it included measures of pair-wise functional connectivity between 90 different regions distributed throughout the brain. The fact that cortico-subcortical systems emerged as a prominent pathology using this completely data-driven analysis provides strong support for the involvement of these systems in internet addiction. Moreover, our findings indicate that internet addiction shares neurobiological characteristics in common with other addictive disorders, and that subcortical regions in particular may represent core sites of brain network pathology. An important note is the view from the National Institute on Drug Abuse that behavioral addictions might be relatively pure models of addiction, taking into account that these conditions are not contaminated by the effects of substances [55]. Whereas the study of many other addiction disorders is invariably confounded by secondary toxicity effects of substance abuse, internet addiction is diagnosed behaviorally and thus provides a more targeted model for studying addiction that is free from long-term drug effects.

In this study, the NBS was used with network size measured based on its extent; that is, the total number of connections comprising the network. This size measure is not suited to detecting focal effects involving single, isolated connections that do not collectively form a network. To test for these kinds of focal between-group differences, the NBS analysis was repeated testing for differences in component mass rather than size. The mass statistic provides greater sensitivity to focal, intense effects than testing for differences in component size. In addition, we also thresholded edge-wise comparisons using the FDR, which will be very sensitive to high-intensity, focal effects. No significant between-group differences were apparent with either the FDR or mass statistics, suggesting that aberrant connectivity in internet addiction encompasses a distributed network involving several cortical and subcortical regions.

Given that brain network properties are known to be sensitive to the choice of parcellation template, we investigated several alternative parcellation schemes in order to assess the reproducibility of any findings to changes in nodal definitions [45], [56], [57]. This enabled us to rule out the possibility that certain findings were merely due to a statistically favorable positioning of nodes, but not reproducible with other well-known parcellations.

In contrast to the decreased strength of functional connectivity, topological parameters revealed no significant group differences. Even when we conducted further exploratory analyses based on less stringent control against type I error, the results indicated possible topological difference mainly involving the occipital lobe, which was unaffected in the NBS analysis. Thus, while internet addiction was associated with a widespread and significant decrease of functional connectivity in cortico-subcortical circuits, this decrease was not associated with a global disruption in brain functional network topology. This study demonstrates that widespread differences in functional connectivity can exist in the absence of alterations in basic topological measures. It may seem remarkable that differences in connectivity strength were so widespread in the absence of any significant topological differences. However, it is important to remark that topology and connectivity strength are distinct properties of the connectome and abnormalities in one need not imply abnormalities in the other. Similar findings have been observed in other disorders [52], [58]. We note however, that group differences in some topological properties trended towards statistical significance. Analysis of a larger sample may afford the necessary power to declare these effects significant. Our results suggest that topological changes may be more subtle than those observed for functional connectivity measures.

Among the 59 connections included in the altered network, 25 were interhemispheric connections and 34 were intrahemispheric, pointing to involvement of long-range as well as short-range connections throughout the brain. Given that internet addiction is a newly recognized mental health condition, with its concept and diagnostic criteria still elusive and undetermined, perhaps it might be surprising to find such an extensively affected network in the brain of these subjects. Recently, Lin et al. (2012) investigated brain white matter integrity in adolescents with internet addiction using diffusion-tensor imaging, and found a widespread decrease in fractional anisotropy (FA) throughout the brain with no area of higher FA in internet addiction group [13]. Such results implicate a possible anatomical basis for the functional disturbances observed in our sample, a hypothesis that could be tested using combined fMRI and diffusion-weighted imaging in the same participants [59].

Regarding the large number of interhemispheric connections found in the altered network, efficient interhemispheric communication has long been thought to be important in brain functions [60]–[63]. However, relatively few neuroimaging studies of addiction have addressed the functional integration between bilateral hemispheres. Recently, Kelly et al. (2011) observed reduced interhemispheric functional connectivity in cocaine-dependent adults [64]. They showed primary involvement of a fronto-parietal network, with a relative sparing of temporal regions, results that parallel our findings. Although the authors discussed the finding mainly as reflecting the long-term effects of chronic cocaine exposure, they also mentioned the possibility that reduced interhemispheric functional connectivity may have preceded the exposure to cocaine as a vulnerability to addiction disorders. Our results suggest that these interhemispheric changes reflect either a vulnerability for addiction disorders or a neural correlate of generic addictive behavior, rather than being secondary to prolonged drug use, given that addiction in our sample was defined in purely behavioral terms. These possibilities could be evaluated by testing for phenotypic similarities between unaffected relatives of individuals with either drug or behavioral addiction disorders.

Interestingly, a similar pattern of decreased resting-state functional connectivity between frontal and parietal regions was reported both in cocaine- and heroin-dependent individuals [64], [65]. In a recent review, Sutherland et al. (2012) suggested that decreased connectivity in the fronto-parietal circuits might be a central component in the impaired cognitive control network of drug-addicted populations [54]. Our finding in internet addiction also supports the notion that decreased functional connectivity between frontal and parietal regions might be a common characteristic across different types of addiction, suggesting the presence of a shared phenotype that is not a secondary consequence of drug use. In addition, a recent investigation of whole-brain functional connectivity in schizophrenia showed prominent fronto-temporal rather than fronto-parietal or fronto-striatal changes [52], consistent with classical pathophysiological models of the disorder [66]. Of course, the most remarkable finding of the present study is that internet addiction was associated with pathology of striatal circuits in particular, a system commonly implicated in other addictive disorders, suggesting a shared neurobiological phenotype. Identifying altered striatal circuits overlapping with those of well-established addictive disorders might be useful for testing whether the addiction model is an appropriate theoretical framework for understanding the disorder [67], [68]. However, whether a relatively stronger impairment of fronto-parietal and fronto-striatal functional connectivity could be largely specific to addiction disorders still remains in question. Future studies directly comparing different disorders are needed in order to establish specificity.

One of the most interesting findings in the current study was the strong involvement of the putamen. This brain structure is known to modulate several neurotransmitters including dopamine, and blunted striatal dopaminergic function has been strongly implicated as one of the key biological mechanisms of addiction disorders [8]. Dopamine is a key modulator of putamen function and may play an important role in the functional connectivity disturbances observed in this study. This is consistent with recent evidence that striatal dopamine transporter and D2 receptor availability is altered in people with internet addiction [6], [7] and that genetic and pharmacologic modulation of brain dopamine levels can exert a profound impact on functional connectivity patterns [69]–[71]. Taking into account these former reports and the proposed neurobiological mechanism of addiction involving blunted striatal dopaminergic function [8], understanding the effects of dopamine on the network identified as showing altered functional connectivity in the present study will represent an important way forward in understanding the neurobiological correlates of internet addiction.

Our finding that the putamen was the most extensively involved subcortical region in the decreased functional network, sparing the caudate nucleus, is also interesting. Both structures are part of the striatum, which in turn is part of the subcortical structures. The putamen is typically considered a brain region associated with motor activity, and has less often been implicated in substance addition than the caudate. Among motor activities, a well-learned sequence of repetitive finger movements has been shown to be associated with activation in the putamen [72]–[76]. People suffering from internet addiction may undergo a far higher frequency of certain behaviors over a long period of time, which include repetitive manipulation of the mouse and keyboard, and these experiences can affect their brain. Therefore, aberrant connectivity stemming from the putamen perhaps indicates a specific characteristic of internet addiction. However, as we did not measure the degree of finger manipulation in our participants, the implication of decreased rather than increased functional connectivity involving the putamen in relation to mouse/keyboard manipulation remains open to future research. Alternatively, the involvement of the putamen in our findings may reflect its role in cognitive processes that are shared with the caudate and which are impaired in addiction, such as reward processing [77], [78].

Another point worthy of discussion is the absence of any increased functional connectivity in the internet addiction group. Although we mainly expected to find decreased functional connectivity in the addiction group, in fact, we did not exclude the possibility of observing increased functional connectivity as well, particularly given the hypothesis that adolescents with internet addiction might show a practice effect due to excessive online activities [79]–[82]. One possible explanation for the negative finding could be that our small sample size lacked the power to detect this practice-related increase in functional connectivity. However, it is not yet fully established whether cognitive performance in certain tasks or severity of certain psychopathologies manifest as decreased or increased functional connectivity [83], [84]. Another consideration should be that the effect of long-term excessive internet use might differentially influence the brain according to subpopulation. For example, a subpopulation called professional online game players engages in intensive internet activities, spends similarly long amounts of time practicing online games and probably performs better in those games than people with internet addiction, and yet seems not to be addicted as evidenced by significantly lower YIAS score [85]. Hence, it could be hypothesized that practice effects in internet activities perhaps manifest differently depending on the individual.

The present study has some important limitations. First, the sample size was quite small, which likely limited our power to detect significant correlations between functional connectivity and YIAS scores. Thus, the current finding needs to be replicated in a larger sample of participants with internet addiction and controls. It is, however, noteworthy that our sample size was on the whole comparable to that of former functional neuroimaging studies of internet addiction. Our sample was unique, as most former studies were based on adults [2]–[7]. Second, the diagnostic criteria for internet addiction are not solidly established yet, though our findings do point to a potential neurobiological basis for this putative disorder. Third, although we excluded comorbid mental disorders using K-SADS-PL, subthreshold-level symptoms of comorbid mental conditions might have still been present. Fourth, collection of a broader range of clinical information such as sleeping habits may have enriched our data and improved our contribution to the literature [86], [87]. Finally, the cross-sectional study design limits the interpretation of a causal relationship between decreased functional connectivity and the development of internet addiction. It should be noted that head motion has emerged as an important confound in functional neuroimaging [38], [88]. Head motion was comprehensively assessed in this study using a range of recently proposed rotational and displacement measures [38]. No significant difference between groups was found for any of the head motion measures considered.

In summary, the results of this study suggest that adolescents with internet addiction display altered brain functional connectivity in the absence of gross disturbances of network topology. The altered network showed an extensive involvement of long-range interhemispheric connections as well as short-range intrahemispheric links throughout the brain. Subcortical brain regions may play an important role in this altered network, particularly the putamen, which showed decreased connections with all three major cerebral lobes involved.

## Supporting Information

Figure S1 **Network of decreased brain functional connectivity in adolescents with internet addiction (using different atlases).**Red dots represent stereotactic centroids of brain regions (nodes) defined by Montreal Neurological Institute (MNI) structural atlas (A) and random parcellation atlas (B), and blue lines represent suprathreshold links (t = 2.1 and 3.0, respectively) comprising the affected network identified with the network-based statistic (NBS) (*p*<0.05, component-wise corrected).
(TIF)

Figure S2 **Proportion of connections affected in internet addiction linking distinct pairs of broad cerebral divisions (detailed for subcortical regions).**The number of links involving each pair of divisions is normalized by total number of pair-wise links.
(TIF)

Table S1 **Local clustering coefficient.**This table shows trend level results with less stringent false positive correction *p*<(1/90) = 0.011; no result survived the standard false discovery rate correction for multiple comparisons.
(DOC)

Table S2 **Local path length.**This table shows trend level results with less stringent false positive correction *p*<(1/90) = 0.011; no result survived the standard false discovery rate correction for multiple comparisons.
(DOC)

## References

[1] Ko CH, Yen JY, Yen CF, Chen CS, Chen CC (2012) The association between Internet addiction and psychiatric disorder: a review of the literature. Eur Psychiatry 27: 1–8. 22153731 10.1016/j.eurpsy.2010.04.011

[2] Dong G, Huang J, Du X (2011) Enhanced reward sensitivity and decreased loss sensitivity in Internet addicts: an fMRI study during a guessing task. J Psychiatr Res 45: 1525–1529. 21764067 10.1016/j.jpsychires.2011.06.017

[3] Han DH, Bolo N, Daniels MA, Arenella L, Lyoo IK, et al (2011) Brain activity and desire for Internet video game play. Compr Psychiatry 52: 88–95. 21220070 10.1016/j.comppsych.2010.04.004 PMC3039876

[4] Han DH, Kim YS, Lee YS, Min KJ, Renshaw PF (2010) Changes in cue-induced, prefrontal cortex activity with video-game play. Cyberpsychol Behav Soc Netw 13: 655–661. 21142990 10.1089/cyber.2009.0327

[5] Ko CH, Liu GC, Hsiao S, Yen JY, Yang MJ, et al (2009) Brain activities associated with gaming urge of online gaming addiction. J Psychiatr Res 43: 739–747. 18996542 10.1016/j.jpsychires.2008.09.012

[6] Kim SH, Baik SH, Park CS, Kim SJ, Choi SW, et al (2011) Reduced striatal dopamine D2 receptors in people with Internet addiction. Neuroreport 22: 407–411. 21499141 10.1097/WNR.0b013e328346e16e

[7] Hou H, Jia S, Hu S, Fan R, Sun W, et al (2012) Reduced striatal dopamine transporters in people with internet addiction disorder. J Biomed Biotechnol 2012: 854524. 22505818 10.1155/2012/854524 PMC3312312

[8] Goldstein RZ, Volkow ND (2011) Dysfunction of the prefrontal cortex in addiction: neuroimaging findings and clinical implications. Nat Rev Neurosci 12: 652–669. 22011681 10.1038/nrn3119 PMC3462342

[9] Goldstein RZ, Craig AD, Bechara A, Garavan H, Childress AR, et al (2009) The neurocircuitry of impaired insight in drug addiction. Trends Cogn Sci 13: 372–380. 19716751 10.1016/j.tics.2009.06.004 PMC2844118

[10] Naqvi NH, Bechara A (2010) The insula and drug addiction: an interoceptive view of pleasure, urges, and decision-making. Brain Struct Funct 214: 435–450. 20512364 10.1007/s00429-010-0268-7 PMC3698865

[11] Zhou Y, Lin FC, Du YS, Qin LD, Zhao ZM, et al (2011) Gray matter abnormalities in Internet addiction: a voxel-based morphometry study. Eur J Radiol 79: 92–95. 19926237 10.1016/j.ejrad.2009.10.025

[12] Yuan K, Qin W, Wang G, Zeng F, Zhao L, et al (2011) Microstructure abnormalities in adolescents with internet addiction disorder. PLoS One 6: e20708. 21677775 10.1371/journal.pone.0020708 PMC3108989

[13] Lin F, Zhou Y, Du Y, Qin L, Zhao Z, et al (2012) Abnormal white matter integrity in adolescents with internet addiction disorder: a tract-based spatial statistics study. PLoS One 7: e30253. 22253926 10.1371/journal.pone.0030253 PMC3256221

[14] Liu J, Gao XP, Osunde I, Li X, Zhou SK, et al (2010) Increased regional homogeneity in internet addiction disorder: a resting state functional magnetic resonance imaging study. Chin Med J (Engl) 123: 1904–1908. 20819576

[15] Yuan K, Qin W, Liu Y, Tian J (2011) Internet addiction: Neuroimaging findings. Commun Integr Biol 4: 637–639. 22448301 10.4161/cib.17871 PMC3306323

[16] Raichle ME, Snyder AZ (2007) A default mode of brain function: a brief history of an evolving idea. Neuroimage 37: 1083–1090; discussion 1097–1089. 17719799 10.1016/j.neuroimage.2007.02.041

[17] Fox MD, Corbetta M, Snyder AZ, Vincent JL, Raichle ME (2006) Spontaneous neuronal activity distinguishes human dorsal and ventral attention systems. Proc Natl Acad Sci U S A 103: 10046–10051. 16788060 10.1073/pnas.0604187103 PMC1480402

[18] Smith SM, Miller KL, Moeller S, Xu J, Auerbach EJ, et al (2012) Temporally-independent functional modes of spontaneous brain activity. Proc Natl Acad Sci U S A 109: 3131–3136. 22323591 10.1073/pnas.1121329109 PMC3286957

[19] Damoiseaux JS, Rombouts SA, Barkhof F, Scheltens P, Stam CJ, et al (2006) Consistent resting-state networks across healthy subjects. Proc Natl Acad Sci U S A 103: 13848–13853. 16945915 10.1073/pnas.0601417103 PMC1564249

[20] Shehzad Z, Kelly AM, Reiss PT, Gee DG, Gotimer K, et al (2009) The resting brain: unconstrained yet reliable. Cereb Cortex 19: 2209–2229. 19221144 10.1093/cercor/bhn256 PMC3896030

[21] Fornito A, Bullmore ET (2012) Connectomic intermediate phenotypes for psychiatric disorders. Front Psychiatry 3: 32. 22529823 10.3389/fpsyt.2012.00032 PMC3329878

[22] Fornito A, Zalesky A, Bassett DS, Meunier D, Ellison-Wright I, et al (2011) Genetic influences on cost-efficient organization of human cortical functional networks. J Neurosci 31: 3261–3270. 21368038 10.1523/JNEUROSCI.4858-10.2011 PMC6623940

[23] Glahn DC, Winkler AM, Kochunov P, Almasy L, Duggirala R, et al (2010) Genetic control over the resting brain. Proc Natl Acad Sci U S A 107: 1223–1228. 20133824 10.1073/pnas.0909969107 PMC2824276

[24] Fox MD, Raichle ME (2007) Spontaneous fluctuations in brain activity observed with functional magnetic resonance imaging. Nat Rev Neurosci 8: 700–711. 17704812 10.1038/nrn2201

[25] Fornito A, Bullmore ET (2010) What can spontaneous fluctuations of the blood oxygenation-level-dependent signal tell us about psychiatric disorders? Current Opinion in Psychiatry 23: 239–249. 20216219 10.1097/YCO.0b013e328337d78d

[26] Bullmore E, Sporns O (2009) Complex brain networks: graph theoretical analysis of structural and functional systems. Nat Rev Neurosci 10: 186–198. 19190637 10.1038/nrn2575

[27] Block JJ (2008) Issues for DSM-V: internet addiction. Am J Psychiatry 165: 306–307. 18316427 10.1176/appi.ajp.2007.07101556

[28] Rubinov M, Sporns O (2010) Complex network measures of brain connectivity: uses and interpretations. Neuroimage 52: 1059–1069. 19819337 10.1016/j.neuroimage.2009.10.003

[29] Xia M, He Y (2011) Magnetic resonance imaging and graph theoretical analysis of complex brain networks in neuropsychiatric disorders. Brain Connect 1: 349–365. 22432450 10.1089/brain.2011.0062

[30] Fornito A, Zalesky A, Pantelis C, Bullmore ET (2012) Schizophrenia, neuroimaging and connectomics. Neuroimage 10.1016/j.neuroimage.2011.12.090 22387165

[31] Gong G, He Y, Evans AC (2011) Brain connectivity: gender makes a difference. Neuroscientist 17: 575–591. 21527724 10.1177/1073858410386492

[32] Widyanto L, McMurran M (2004) The psychometric properties of the internet addiction test. Cyberpsychol Behav 7: 443–450. 15331031 10.1089/cpb.2004.7.443

[33] Kaufman J, Birmaher B, Brent D, Rao U, Flynn C, et al (1997) Schedule for Affective Disorders and Schizophrenia for School-Age Children-Present and Lifetime Version (K-SADS-PL): initial reliability and validity data. J Am Acad Child Adolesc Psychiatry 36: 980–988. 9204677 10.1097/00004583-199707000-00021

[34] Kim YS, Cheon KA, Kim BN, Chang SA, Yoo HJ, et al (2004) The reliability and validity of Kiddie-Schedule for Affective Disorders and Schizophrenia-Present and Lifetime Version- Korean version (K-SADS-PL-K). Yonsei Med J 45: 81–89. 15004873 10.3349/ymj.2004.45.1.81

[35] Christakis DA (2010) Internet addiction: a 21st century epidemic? BMC Med 8: 61. 20955578 10.1186/1741-7015-8-61 PMC2972229

[36] Flisher C (2010) Getting plugged in: an overview of internet addiction. J Paediatr Child Health 46: 557–559. 20979347 10.1111/j.1440-1754.2010.01879.x

[37] Chao-Gan Y, Yu-Feng Z (2010) DPARSF: A MATLAB Toolbox for "Pipeline" Data Analysis of Resting-State fMRI. Front Syst Neurosci 4: 13. 20577591 10.3389/fnsys.2010.00013 PMC2889691

[38] Van Dijk KR, Sabuncu MR, Buckner RL (2012) The influence of head motion on intrinsic functional connectivity MRI. Neuroimage 59: 431–438. 21810475 10.1016/j.neuroimage.2011.07.044 PMC3683830

[39] Wilke M, Holland SK, Altaye M, Gaser C (2008) Template-O-Matic: a toolbox for creating customized pediatric templates. Neuroimage 41: 903–913. 18424084 10.1016/j.neuroimage.2008.02.056

[40] Tzourio-Mazoyer N, Landeau B, Papathanassiou D, Crivello F, Etard O, et al (2002) Automated anatomical labeling of activations in SPM using a macroscopic anatomical parcellation of the MNI MRI single-subject brain. Neuroimage 15: 273–289. 11771995 10.1006/nimg.2001.0978

[41] Zalesky A, Fornito A, Bullmore ET (2010) Network-based statistic: identifying differences in brain networks. Neuroimage 53: 1197–1207. 20600983 10.1016/j.neuroimage.2010.06.041

[42] Zalesky A, Cocchi L, Fornito A, Murray MM, Bullmore E (2012) Connectivity differences in brain networks. Neuroimage 60: 1055–1062. 22273567 10.1016/j.neuroimage.2012.01.068

[43] Nichols TE, Holmes AP (2002) Nonparametric permutation tests for functional neuroimaging: A primer with examples. Human Brain Mapping 15: 1–25. 11747097 10.1002/hbm.1058 PMC6871862

[44] Wig GS, Schlaggar BL, Petersen SE (2011) Concepts and principles in the analysis of brain networks. Ann N Y Acad Sci 1224: 126–146. 21486299 10.1111/j.1749-6632.2010.05947.x

[45] Zalesky A, Fornito A, Harding IH, Cocchi L, Yucel M, et al (2010) Whole-brain anatomical networks: Does the choice of nodes matter? Neuroimage 50: 970–983. 20035887 10.1016/j.neuroimage.2009.12.027

[46] Bassett DS, Bullmore ET (2009) Human brain networks in health and disease. Curr Opin Neurol 22: 340–347. 19494774 10.1097/WCO.0b013e32832d93dd PMC2902726

[47] Guye M, Bettus G, Bartolomei F, Cozzone PJ (2010) Graph theoretical analysis of structural and functional connectivity MRI in normal and pathological brain networks. MAGMA 23: 409–421. 20349109 10.1007/s10334-010-0205-z

[48] He Y, Evans A (2010) Graph theoretical modeling of brain connectivity. Curr Opin Neurol 23: 341–350. 20581686 10.1097/WCO.0b013e32833aa567

[49] Bassett DS, Gazzaniga MS (2011) Understanding complexity in the human brain. Trends Cogn Sci 15: 200–209. 21497128 10.1016/j.tics.2011.03.006 PMC3170818

[50] Maslov S, Sneppen K (2002) Specificity and stability in topology of protein networks. Science 296: 910–913. 11988575 10.1126/science.1065103

[51] Genovese CR, Lazar NA, Nichols T (2002) Thresholding of statistical maps in functional neuroimaging using the false discovery rate. Neuroimage 15: 870–878. 11906227 10.1006/nimg.2001.1037

[52] Fornito A, Yoon J, Zalesky A, Bullmore ET, Carter CS (2011) General and specific functional connectivity disturbances in first-episode schizophrenia during cognitive control performance. Biol Psychiatry 70: 64–72. 21514570 10.1016/j.biopsych.2011.02.019 PMC4015465

[53] Lynall ME, Bassett DS, Kerwin R, McKenna PJ, Kitzbichler M, et al (2010) Functional connectivity and brain networks in schizophrenia. J Neurosci 30: 9477–9487. 20631176 10.1523/JNEUROSCI.0333-10.2010 PMC2914251

[54] Sutherland MT, McHugh MJ, Pariyadath V, Stein EA (2012) Resting state functional connectivity in addiction: Lessons learned and a road ahead. Neuroimage 62: 2281–2295. 22326834 10.1016/j.neuroimage.2012.01.117 PMC3401637

[55] Shaw M, Black DW (2008) Internet addiction: definition, assessment, epidemiology and clinical management. CNS Drugs 22: 353–365. 18399706 10.2165/00023210-200822050-00001

[56] Wang J, Wang L, Zang Y, Yang H, Tang H, et al (2009) Parcellation-dependent small-world brain functional networks: a resting-state fMRI study. Hum Brain Mapp 30: 1511–1523. 18649353 10.1002/hbm.20623 PMC6870680

[57] Fornito A, Zalesky A, Bullmore ET (2010) Network scaling effects in graph analytic studies of human resting-state FMRI data. Front Syst Neurosci 4: 22. 20592949 10.3389/fnsys.2010.00022 PMC2893703

[58] Cocchi L, Bramati IE, Zalesky A, Furukawa E, Fontenelle L, et al (2012) Altered functional brain connectivity in a non-clinical sample of young adults with Attention-Deficit/Hyperactivity Disorder. J Neurosci 10.1523/JNEUROSCI.3272-12.2012 PMC6621678 23223295

[59] Honey CJ, Sporns O, Cammoun L, Gigandet X, Thiran JP, et al (2009) Predicting human resting-state functional connectivity from structural connectivity. Proc Natl Acad Sci U S A 106: 2035–2040. 19188601 10.1073/pnas.0811168106 PMC2634800

[60] Anderson JS, Druzgal TJ, Froehlich A, DuBray MB, Lange N, et al (2011) Decreased interhemispheric functional connectivity in autism. Cereb Cortex 21: 1134–1146. 20943668 10.1093/cercor/bhq190 PMC3077433

[61] Clarke AR, Barry RJ, Heaven PC, McCarthy R, Selikowitz M, et al (2008) EEG coherence in adults with attention-deficit/hyperactivity disorder. Int J Psychophysiol 67: 35–40. 18029040 10.1016/j.ijpsycho.2007.10.001

[62] Pettigrew JD, Miller SM (1998) A 'sticky' interhemispheric switch in bipolar disorder? Proc Biol Sci 265: 2141–2148. 9872002 10.1098/rspb.1998.0551 PMC1689515

[63] Spencer KM, Nestor PG, Niznikiewicz MA, Salisbury DF, Shenton ME, et al (2003) Abnormal neural synchrony in schizophrenia. J Neurosci 23: 7407–7411. 12917376 10.1523/JNEUROSCI.23-19-07407.2003 PMC2848257

[64] Kelly C, Zuo XN, Gotimer K, Cox CL, Lynch L, et al (2011) Reduced interhemispheric resting state functional connectivity in cocaine addiction. Biol Psychiatry 69: 684–692. 21251646 10.1016/j.biopsych.2010.11.022 PMC3056937

[65] Yuan K, Qin W, Dong M, Liu J, Sun J, et al (2010) Gray matter deficits and resting-state abnormalities in abstinent heroin-dependent individuals. Neurosci Lett 482: 101–105. 20621162 10.1016/j.neulet.2010.07.005

[66] Fletcher P, McKenna PJ, Friston KJ, Frith CD, Dolan RJ (1999) Abnormal cingulate modulation of fronto-temporal connectivity in schizophrenia. Neuroimage 9: 337–342. 10075903 10.1006/nimg.1998.0411

[67] Choi JS, Shin YC, Jung WH, Jang JH, Kang DH, et al (2012) Altered brain activity during reward anticipation in pathological gambling and obsessive-compulsive disorder. PLoS One 7: e45938. 23029329 10.1371/journal.pone.0045938 PMC3447818

[68] Tomasi D, Volkow ND (2012) Striatocortical pathway dysfunction in addiction and obesity: differences and similarities. Crit Rev Biochem Mol Biol 10.3109/10409238.2012.735642 PMC3557663 23173916

[69] Cole DM, Oei NY, Soeter RP, Both S, van Gerven JM, et al (2012) Dopamine-Dependent Architecture of Cortico-Subcortical Network Connectivity. Cereb Cortex 10.1093/cercor/bhs136 22645252

[70] Gordon EM, Stollstorff M, Devaney JM, Bean S, Vaidya CJ (2011) Effect of Dopamine Transporter Genotype on Intrinsic Functional Connectivity Depends on Cognitive State. Cereb Cortex 10.1093/cercor/bhr305 PMC3412445 22047966

[71] Rieckmann A, Karlsson S, Fischer H, Backman L (2011) Caudate dopamine D1 receptor density is associated with individual differences in frontoparietal connectivity during working memory. J Neurosci 31: 14284–14290. 21976513 10.1523/JNEUROSCI.3114-11.2011 PMC6623648

[72] Lehericy S, Bardinet E, Tremblay L, Van de Moortele PF, Pochon JB, et al (2006) Motor control in basal ganglia circuits using fMRI and brain atlas approaches. Cereb Cortex 16: 149–161. 15858164 10.1093/cercor/bhi089

[73] Jenkins IH, Brooks DJ, Nixon PD, Frackowiak RS, Passingham RE (1994) Motor sequence learning: a study with positron emission tomography. J Neurosci 14: 3775–3790. 8207487 10.1523/JNEUROSCI.14-06-03775.1994 PMC6576955

[74] Jueptner M, Frith CD, Brooks DJ, Frackowiak RS, Passingham RE (1997) Anatomy of motor learning. II. Subcortical structures and learning by trial and error. J Neurophysiol 77: 1325–1337. 9084600 10.1152/jn.1997.77.3.1325

[75] Roland PE, Meyer E, Shibasaki T, Yamamoto YL, Thompson CJ (1982) Regional cerebral blood flow changes in cortex and basal ganglia during voluntary movements in normal human volunteers. J Neurophysiol 48: 467–480. 6981690 10.1152/jn.1982.48.2.467

[76] Shibasaki H, Sadato N, Lyshkow H, Yonekura Y, Honda M, et al (1993) Both primary motor cortex and supplementary motor area play an important role in complex finger movement. Brain 116 (Pt 6): 1387–1398. 8293277 10.1093/brain/116.6.1387

[77] Volkow ND, Fowler JS, Wang GJ (2003) The addicted human brain: insights from imaging studies. J Clin Invest 111: 1444–1451. 12750391 10.1172/JCI18533 PMC155054

[78] Yuan K, Qin W, Liu J, Guo Q, Dong M, et al (2010) Altered small-world brain functional networks and duration of heroin use in male abstinent heroin-dependent individuals. Neurosci Lett 477: 37–42. 20417253 10.1016/j.neulet.2010.04.032

[79] Di X, Zhu S, Jin H, Wang P, Ye Z, et al (2012) Altered resting brain function and structure in professional badminton players. Brain Connect 2: 225–233. 22840241 10.1089/brain.2011.0050 PMC3621728

[80] Duan X, Liao W, Liang D, Qiu L, Gao Q, et al (2012) Large-scale brain networks in board game experts: insights from a domain-related task and task-free resting state. PLoS One 7: e32532. 22427852 10.1371/journal.pone.0032532 PMC3299676

[81] Ma L, Narayana S, Robin DA, Fox PT, Xiong J (2011) Changes occur in resting state network of motor system during 4 weeks of motor skill learning. Neuroimage 58: 226–233. 21689765 10.1016/j.neuroimage.2011.06.014 PMC3144281

[82] Martinez K, Solana AB, Burgaleta M, Hernandez-Tamames JA, Alvarez-Linera J, et al (2012) Changes in resting-state functionally connected parietofrontal networks after videogame practice. Hum Brain Mapp 10.1002/hbm.22129 PMC6870012 22807280

[83] Zhou Y, Liang M, Tian L, Wang K, Hao Y, et al (2007) Functional disintegration in paranoid schizophrenia using resting-state fMRI. Schizophr Res 97: 194–205. 17628434 10.1016/j.schres.2007.05.029

[84] Whitfield-Gabrieli S, Thermenos HW, Milanovic S, Tsuang MT, Faraone SV, et al (2009) Hyperactivity and hyperconnectivity of the default network in schizophrenia and in first-degree relatives of persons with schizophrenia. Proc Natl Acad Sci U S A 106: 1279–1284. 19164577 10.1073/pnas.0809141106 PMC2633557

[85] Han DH, Lyoo IK, Renshaw PF (2012) Differential regional gray matter volumes in patients with on-line game addiction and professional gamers. J Psychiatr Res 46: 507–515. 22277302 10.1016/j.jpsychires.2012.01.004 PMC4632992

[86] De Havas JA, Parimal S, Soon CS, Chee MW (2012) Sleep deprivation reduces default mode network connectivity and anti-correlation during rest and task performance. Neuroimage 59: 1745–1751. 21872664 10.1016/j.neuroimage.2011.08.026

[87] Killgore WD, Schwab ZJ, Weiner MR (2012) Self-reported nocturnal sleep duration is associated with next-day resting state functional connectivity. Neuroreport 23: 741–745. 22872066 10.1097/WNR.0b013e3283565056

[88] Power JD, Barnes KA, Snyder AZ, Schlaggar BL, Petersen SE (2012) Spurious but systematic correlations in functional connectivity MRI networks arise from subject motion. Neuroimage 59: 2142–2154. 22019881 10.1016/j.neuroimage.2011.10.018 PMC3254728

