# Supplementary figures and images for: Decreased Functional Brain Connectivity in Adolescents with Internet Addiction
Source: PLoS One. 2013 Feb 25;8(2):e57831. doi: 10.1371/journal.pone.0057831 (PMC3581468; doi:10.1371/journal.pone.0057831)

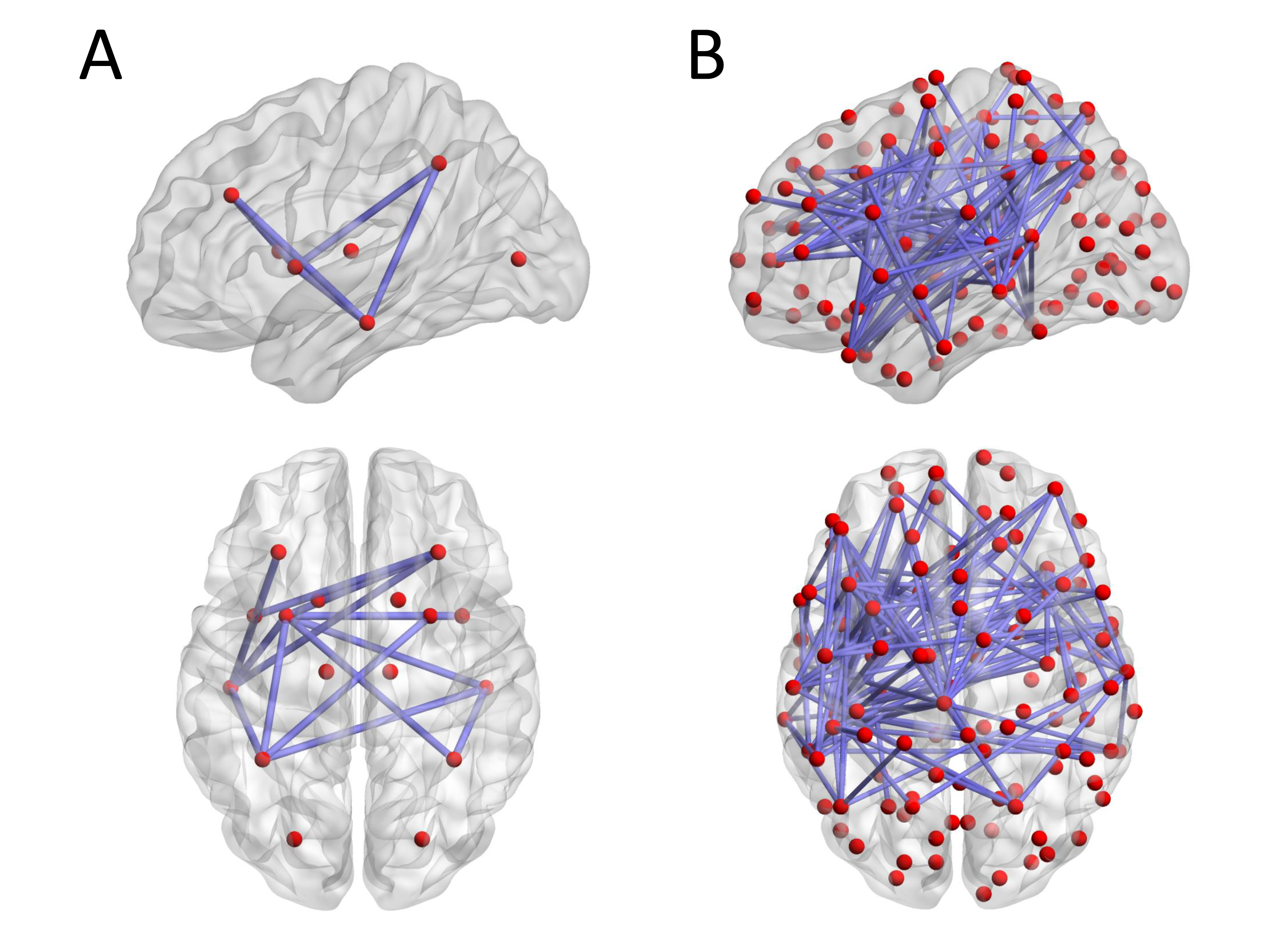

Supplement: Figure S1 — Network of decreased brain functional connectivity in adolescents with internet addiction (using different atlases).Red dots represent stereotactic centroids of brain regions (nodes) defined by Montreal Neurological Institute (MNI) structural atlas (A) and random parcellation atlas (B), and blue lines represent suprathreshold links (t = 2.1 and 3.0, respectively) comprising the affected network identified with the network-based statistic (NBS) (p<0.05, component-wise corrected). (TIF) [file pone.0057831.s001.tif]

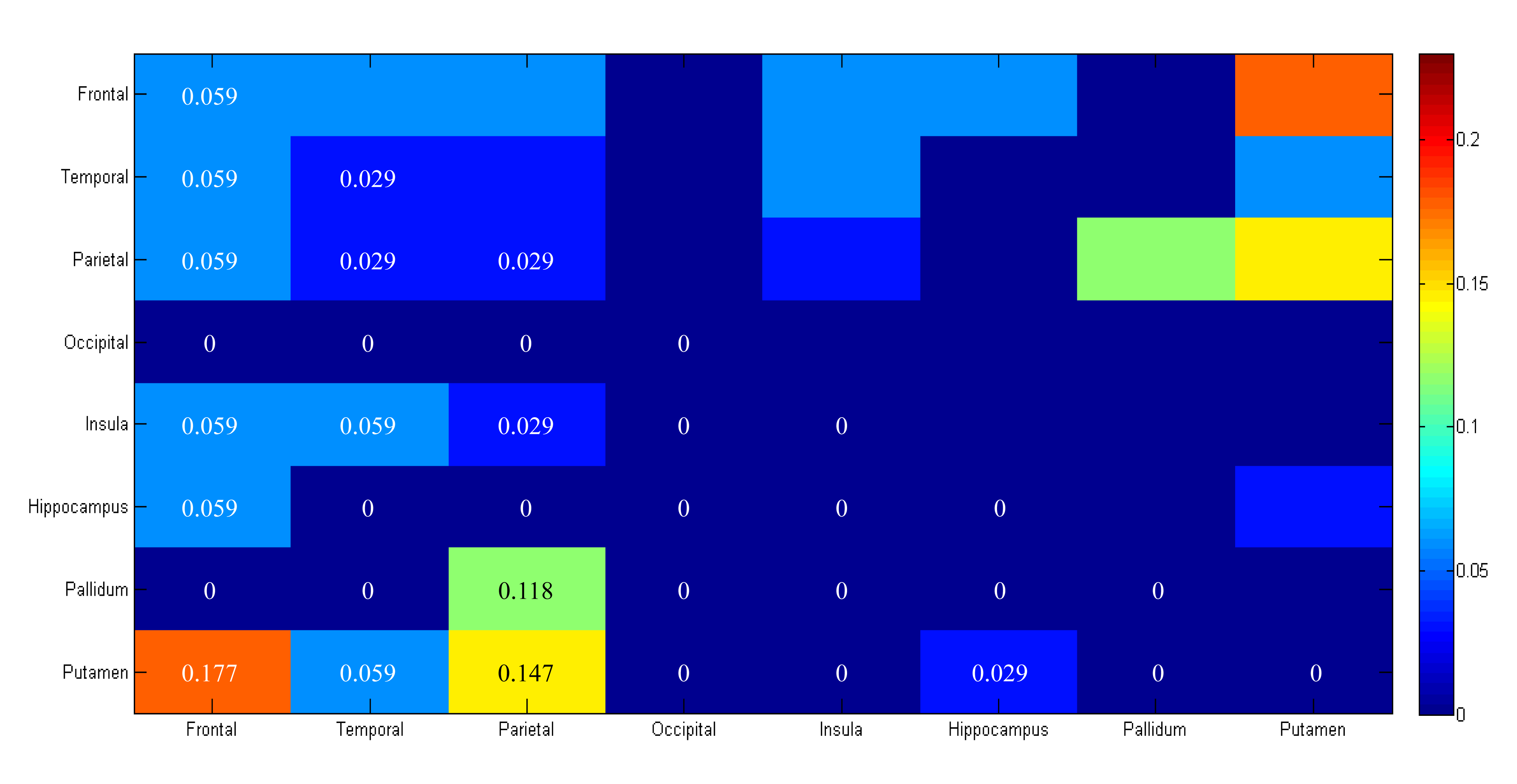

Supplement: Figure S2 — Proportion of connections affected in internet addiction linking distinct pairs of broad cerebral divisions (detailed for subcortical regions).The number of links involving each pair of divisions is normalized by total number of pair-wise links. (TIF) [file pone.0057831.s002.tif]
