# Supplementary material for: Decreased Functional Brain Connectivity in Adolescents with Internet Addiction
Source: PLoS One. 2013 Feb 25;8(2):e57831. doi: 10.1371/journal.pone.0057831 (PMC3581468; doi:10.1371/journal.pone.0057831)
Supplement: Table S1 — Local clustering coefficient.This table shows trend level results with less stringent false positive correction p<(1/90) = 0.011; no result survived the standard false discovery rate correction for multiple comparisons. (DOC) [file pone.0057831.s003.doc]

| Density (%) | Comparison | Side | Node | Mean±SD | | P-value |
| --- | --- | --- | --- | --- | --- | --- |
|  |  |  |  | Internet | Control |  |
| 0.10 | Internet > Control | Left | Olfactory cortex | 0.50±0.43 | 0.05±0.11 | 0.0026 |
|  | Internet > Control | Right | Olfactory cortex | 0.45±0.42 | 0.06±0.20 | 0.0109 |
|  | Internet < Control | Left | Pallidum | 0.35±0.30 | 0.75±0.34 | 0.0075 |
| 0.20 | Internet < Control | Left | Cuneus | 0.71±0.15 | 0.87±0.09 | 0.0051 |
| 0.25 | Internet < Control | Left | Cuneus | 0.73±0.15 | 0.87±0.09 | 0.0099 |
| 0.30 | No significant node for clustering coefficient | | | | | |
