# Supplementary material for: Decreased Functional Brain Connectivity in Adolescents with Internet Addiction
Source: PLoS One. 2013 Feb 25;8(2):e57831. doi: 10.1371/journal.pone.0057831 (PMC3581468; doi:10.1371/journal.pone.0057831)
Supplement: Table S2 — Local path length.This table shows trend level results with less stringent false positive correction p<(1/90) = 0.011; no result survived the standard false discovery rate correction for multiple comparisons. (DOC) [file pone.0057831.s004.doc]

| Density (%) | Comparison | Side | Node | Mean±SD | | P-value |
| --- | --- | --- | --- | --- | --- | --- |
|  |  |  |  | Internet | Control |  |
| 0.10 | No significant node for path length | | | | | |
| 0.20 | Internet < Control | Left | Superior occipital gyrus | 0.69±0.11 | 0.76±0.13 | 0.0073 |
|  | Internet < Control | Right | Inferior occipital gyrus | 0.67±0.13 | 0.81±0.13 | 0.0032 |
| 0.25 | Internet < Control | Left | Superior occipital gyrus | 0.70±0.11 | 0.79±0.11 | 0.0041 |
|  | Internet < Control | Right | Inferior occipital gyrus | 0.70±0.12 | 0.79±0.13 | 0.0089 |
| 0.30 | Internet < Control | Left | Superior occipital gyrus | 0.70±0.09 | 0.78±0.11 | 0.0016 |
|  | Internet < Control | Right | Inferior occipital gyrus | 0.69±0.09 | 0.79±0.11 | 0.0047 |
